# The Tip of the VgrG Spike Is Essential to Functional Type VI Secretion System Assembly in *Acinetobacter baumannii*

**DOI:** 10.1128/mBio.02761-19

**Published:** 2020-01-14

**Authors:** Juvenal Lopez, Pek Man Ly, Mario F. Feldman

**Affiliations:** a Department of Molecular Microbiology, Washington University School of Medicine in St. Louis, St. Louis, Missouri, USA; University of Georgia

**Keywords:** T6SS, T6SS assembly, VgrG, bacterial competition, contractile injection system, secretion systems, type VI secretion system

## Abstract

The type VI secretion system (T6SS) is a critical weapon in bacterial warfare between Gram-negative bacteria. Although invaluable for niche establishment, this machine represents an energetic burden to its host bacterium. Acinetobacter baumannii is an opportunistic pathogen that poses a serious threat to public health due to its high rates of multidrug resistance. In some A. baumannii strains, the T6SS is transcriptionally downregulated by large multidrug resistance plasmids. Other strains, such as the clinical isolate AbCAN2, express T6SS-related genes but lack T6SS activity under laboratory conditions, despite not harboring these plasmids. This suggests that alternative mechanisms exist to repress the T6SS. Here, we used a transposon mutagenesis approach in AbCAN2 to identify novel T6SS repressors. Our screen revealed that the T6SS of this strain is inhibited by a homolog of VgrG, an essential structural component of all T6SSs reported to date. We named this protein inhibitory VgrG (VgrGi). Biochemical and *in silico* analyses demonstrated that the unprecedented inhibitory capability of VgrGi is due to a single amino acid mutation in a widely conserved C-terminal domain of unknown function, DUF2345. We also show that unlike in other bacteria, the C terminus of VgrG is essential for functional T6SS assembly in A. baumannii. Our study provides insight into the architectural requirements underlying functional assembly of the T6SS of A. baumannii. We propose that T6SS-inactivating point mutations are beneficial to the host bacterium, since they eliminate the energy cost associated with maintaining a functional T6SS, which appears to be unnecessary for A. baumannii virulence.

## Abstract (precis)

Despite the clinical relevance of A. baumannii, little is known about its fundamental biology. Here, we show that a single amino acid mutation in VgrG, a critical T6SS structural protein, abrogates T6SS function. Given that this mutation was found in a clinical isolate, we propose that the T6SS of A. baumannii is probably not involved in virulence; this idea is supported by multiple genomic analyses showing that the majority of clinical A. baumannii strains lack proteins essential to the T6SS. We also show that, unlike in other species, the C terminus of VgrG is a unique architectural requirement for functional T6SS assembly in A. baumannii, suggesting that over evolutionary time, bacteria have developed changes to their T6SS architecture, leading to specialized systems.

## INTRODUCTION

Bacterial life involves constant interactions between diverse bacterial species. These bacterium-bacterium interactions underlie the formation of complex bacterial communities, which are the subject of constant compositional changes due to factors, such as nutrient availability and interbacterial antagonism (1, 2). Several seminal studies have shown that it is essential for bacterial pathogens to outcompete the normal host microbiota to establish a niche and cause disease (3–5). Interbacterial competition has also been shown to facilitate the exchange of genetic material, thus promoting the dissemination of antibiotic resistance genes and virulence factors (6–9). Understanding how bacteria compete with one another provides insight into a critical aspect of bacterial life.

The type VI secretion system (T6SS) mediates interbacterial competition between Gram-negative bacteria (2, 10, 11). It is composed of a minimum of thirteen conserved structural proteins (TssA to TssM), most of which are encoded in a single locus (12). These constituents assemble into a trans-envelope, bacteriophage tail-like structure that delivers toxic effector proteins into adjacent bacterial cells (13, 14). The “tail” is a cytosolic complex composed of a tube of Hcp (or TssD) hexamers topped with a spike of three VgrG (or TssI) proteins and, in some cases, a single PAAR protein. Hcp, VgrG, and PAAR bind antibacterial T6SS effectors, which target essential cell structures, such as the cell wall, genetic material, or the cell membrane (15, 16). The spiked tube is in turn encompassed by a contractile sheath comprised of TssB and TssC. When the sheath contracts, it propels the effector-loaded spiked tube outward from the attacking cell and into adjacent cells. After its contraction, the sheath is disassembled by the ATPase ClpV (or TssH) to enable sheath constituents to be reused in subsequent T6SS assemblies (17).

The dynamic T6SS tail is built from a multimeric protein complex known as the baseplate, and it is anchored to the bacterial envelope by a membrane complex (18, 19). The membrane complex is composed of three membrane-associated proteins: TssJ, TssM, and TssL (19). In contrast, the baseplate forms in the cytoplasm, beginning with the trimerization of VgrG and the subsequent assembly of wedge proteins (TssE-G and TssK) around the VgrG hub (20–22). Following its assembly, the baseplate interacts with the membrane complex (18, 22, 23), and the tube and sheath assemble at the N terminus of VgrG in a highly coordinated process facilitated by TssA (18, 24).

The T6SS is energetically costly (25). Thus, many T6SS-encoding bacteria tightly regulate their T6SS at transcriptional, posttranscriptional, and/or posttranslational levels (26, 27). The complex regulatory networks governing T6SS activity enable bacteria to respond according to environmental signals, including: temperature, pH, cation/nutrient availability, osmolarity, and membrane perturbation (26, 28–36). For instance, the antibacterial T6SS of Pseudomonas aeruginosa (HSI-I), regarded as a defensive T6SS, remains silenced unless P. aeruginosa perceives an attack from a competing bacterium (37). When an attack is perceived, a posttranslational threonine phosphorylation pathway activates the forkhead-associated domain-containing protein Fha1, ultimately resulting in the assembly of a functional T6SS in the location of the perceived attack (37). Thus, P. aeruginosa overcomes the high energy cost of the T6SS by preventing futile T6SS attacks in the absence of competing bacteria. However, the mechanisms underlying T6SS regulation in other less-studied medically relevant bacteria remain poorly understood.

Acinetobacter baumannii is a Gram-negative bacterial pathogen with alarming rates of multidrug resistance and mortality (38). We have previously shown that A. baumannii strains encode a single, highly conserved T6SS locus (39). Despite the notable genetic conservation, diverse A. baumannii strains possess varying levels of Hcp secretion, the hallmark of T6SS activity. One subset of strains secretes Hcp under standard laboratory conditions, indicating that they possess a constitutively active (or offensive) T6SS. A second subset of strains possess a silenced T6SS because they harbor a family of multidrug-resistance plasmids named large conjugative plasmids (LCPs), which transcriptionally repress the T6SS locus (40–42). The final subset of A. baumannii strains expresses T6SS-related genes and does not harbor an LCP but does not secrete Hcp under standard laboratory conditions (39, 43–45). These strains are expected to regulate their T6SS by as-yet-uncharacterized mechanisms. The clinical A. baumannii strain CAN2 (AbCAN2, formerly named Ab1225) expresses Hcp but does not secrete it under standard laboratory conditions (39). Here, we report the whole-genome sequence of AbCAN2 and determine that it encodes all genes necessary for a functional T6SS and lacks an LCP. Using a transposon mutagenesis approach, we identified a VgrG homolog as an unexpected inhibitor of the T6SS of AbCAN2. Our bioinformatics and biochemical analyses provide insight into the characteristics that differentiate an inhibitory VgrG from a canonical VgrG. Furthermore, we demonstrate a previously unappreciated role for the C terminus of VgrG proteins in T6SS assembly in A. baumannii.

## RESULTS

### Clinical strain AbCAN2 encodes a T6SS locus.

**FIG 1:**
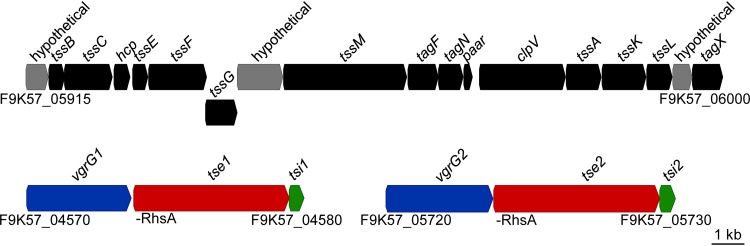
AbCAN2 encodes conserved T6SS-related genes. A schematic of the T6SS locus and *vgrG* gene clusters encoded by AbCAN2 is shown. Locus tags, as well as functional domains identified in *tse1* and *tse2*, are indicated.

AbCAN2 is a coccygeal isolate collected at the University of Alberta Hospital, Canada, with an inactive T6SS under standard laboratory conditions (39). To gain a better understanding of why the T6SS is inactive in this strain, we determined the whole-genome sequence of AbCAN2 (GenBank accession number CP045428). We found that this strain does not harbor an LCP. In addition, it encodes all genes required for a functional T6SS. In fact, the T6SS locus of AbCAN2 is syntenic with that of other A. baumannii strains, including the lab strain A. baumannii ATCC 17978 (Ab17978) (Fig. 1) (39). The T6SS genes of AbCAN2 and Ab17978 present remarkably high levels of sequence conservation, ranging from 98 to 100% nucleotide identity (see Table S1 in the supplemental material). Besides encoding 12 of the 13 structural T6SS proteins conserved among *Proteobacteria* (Acinetobacter species lack a TssJ homolog [39]), the T6SS locus of AbCAN2 also contains genes coding for accessory proteins TagF, TagN, PAAR, and TagX. In addition, the T6SS locus harbors genes that code for three hypothetical proteins that are conserved among Acinetobacter species, some of which have been shown to be essential for T6SS function (46, 47). The specific roles of these proteins are yet to be elucidated.

10.1128/mBio.02761-19.1 TABLE S1 Comparison between T6SS-related genes of AbCAN2 and Ab17978. Download Table S1, DOCX file, 0.01 MB. Copyright © 2020 Lopez et al. 2020 Lopez et al. This content is distributed under the terms of the Creative Commons Attribution 4.0 International license.

AbCAN2 also encodes two *vgrG* gene clusters located remotely from its T6SS locus (Fig. 1). We arbitrarily named the VgrGs of each cluster VgrG1 and VgrG2. Each VgrG is encoded upstream of an open reading frame (ORF) containing a predicted rearrangement hotspot A (RhsA) domain. T6SS effectors of the RhsA family have been previously reported to possess DNase activity (48–50); thus, it is possible that the proteins coded by these ORFs possess nuclease activity. Given their genetic proximity to *vgrG1* and *vgrG2*, we expect these proteins to be T6SS effectors. Thus, we named these ORFs type 6 effector 1 (*tse1*) and *tse2*, respectively. Downstream of each *tse1* and *tse2* is an ORF that we predict to be the cognate immunity proteins of these effectors. Immunity proteins are commonly encoded downstream of their cognate effector, and they inactive their cognate effector by specifically binding it and obstructing its active site (10). Thus, we named these ORFs type six immunity 1 (*tsi1*) and *tsi2*, respectively.

### A VgrG homolog inhibits the T6SS of AbCAN2.

**FIG 2:**
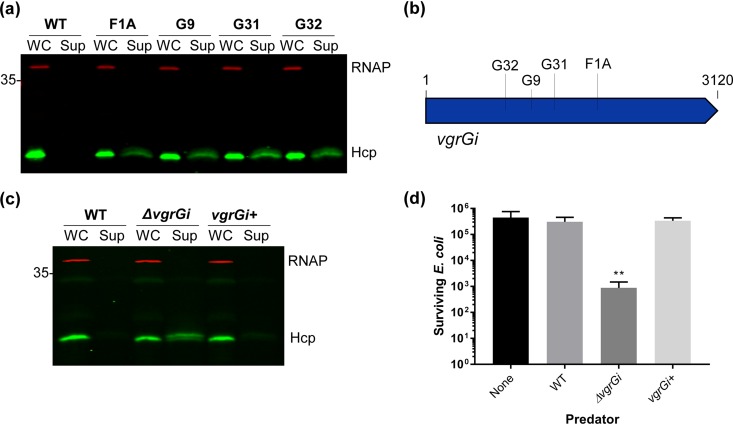
VgrGi inhibits the T6SS of AbCAN2. (a) Western blot of OD-normalized whole-cell (WC) and supernatant (Sup) fractions of wild-type (WT) or unique transposon insertion mutants of AbCAN2 probing for Hcp expression and secretion. (b) Schematic of transposon insertion sites within *vgrGi.* G32, A853; G9, A1108; G31, A1362; F1A, A1846. (c) Western blot probing for Hcp expression and secretion in AbCAN2 WT, *ΔvgrGi*, or the complemented strain (*vgrGi^+^*). For panels a and c, RNAP was included as a lysis and loading control. (d) Cumulative data (means ± the standard deviations) of three independent T6SS killing assays, each with technical duplicates. Surviving E. coli MG1655R pBAV-*gfp* were enumerated after a 3.5-h incubation with the indicated AbCAN2 predator strains at a 10:1 predator/prey ratio. **, *P* = 0.001 (determined by one-way analysis of variance [ANOVA], followed by Dunnett’s multiple-comparison test). For panels c and d, WT and *ΔvgrGi* strains harboring empty vector pWH1266 are shown. The Western blots shown are representative of three independent experiments.

Our finding that AbCAN2 lacks T6SS activity despite encoding all genes required for T6SS assembly and not harboring an LCP led us to hypothesize that AbCAN2 encodes a novel T6SS repressor. To identify the putative novel repressor of the T6SS, we generated a transposon mutant library and screened mutants for Hcp secretion, using our high-throughput Hcp colony blot assay (see Materials and Methods) (51). We hypothesized that genetic disruption of a T6SS inhibitor would result in appreciable T6SS activity in a mutant strain. Of the ∼3,000 colonies screened, we detected Hcp in culture supernatants of four unique mutant strains, indicating that these transposon mutants possess an active T6SS (Fig. 2a). Surprisingly, the gene disrupted in all four mutant strains was *vgrG1* (Fig. 2b). VgrG is a conserved structural component of all T6SSs reported to date. It is a modular protein containing N-terminal domains structurally similar to spike complex proteins of bacteriophage contractile tails (gp27 and gp5), as well as C-terminal DUF2345 and transthyretin (TT)-like domains, whose roles are yet to be fully understood (20, 21, 52–54). VgrGs are widely regarded as essential to T6SS assembly and function, being implicated in roles such as baseplate formation, proper Hcp tube assembly, and effector delivery (18, 22, 24, 55). Thus, our result that a VgrG homolog inhibits the T6SS is against the current paradigm of VgrG function. To confirm our results, we generated a clean deletion mutant of *vgrG1* (hereafter referred to as *vgrGi* for inhibitory VgrG) and compared its T6SS activity to that of wild-type (WT) AbCAN2, using Hcp secretion and bacterial killing assays. Consistent with our previous result, Hcp was detected in the supernatant fraction of *ΔvgrGi* but not WT AbCAN2 or the complemented strain (*vgrGi^+^*), indicating that VgrGi inhibits the T6SS of AbCAN2 (Fig. 2c and Fig. S1a). Similarly, AbCAN2*ΔvgrGi*, but not WT or *vgrGi^+^*, demonstrated significant levels of interbacterial killing when coincubated with E. coli (Fig. 2d). Altogether, our data indicate that VgrGi inhibits T6SS activity in AbCAN2.

10.1128/mBio.02761-19.3 FIG S1 VgrGi inhibits the T6SS of AbCAN2, while VgrG2 is essential for bacterial killing. (a) Western blot of OD-normalized whole cell fractions of the indicated AbCAN2 strains probing for VgrGi-His expression. RNAP was included as a loading control. The Western blot shown is representative of three independent experiments. (b) Cumulative data (means ± the standard deviations) from three independent T6SS killing assays, each with technical duplicates. Surviving E. coli HB101 pWH1266 were enumerated after a 3.5-h incubation with the indicated AbCAN2 predator strains at a 10:1 predator/prey ratio. ***, *P* < 0.001 (determined by one-way ANOVA, followed by Dunnett’s multiple-comparison test). Download FIG S1, TIF file, 1.0 MB. Copyright © 2020 Lopez et al. 2020 Lopez et al. This content is distributed under the terms of the Creative Commons Attribution 4.0 International license.

### VgrGi acts independently to inhibit the T6SS.

**FIG 3:**
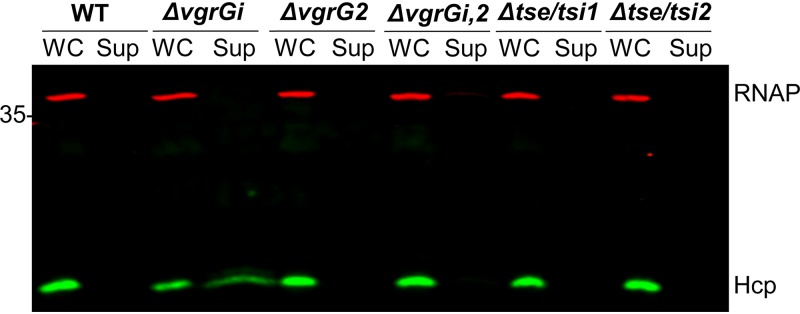
Additional VgrG-related proteins are not involved in T6SS inhibition. Western blot of OD-normalized whole-cell (WC) and supernatant (Sup) fractions of wild-type (WT) AbCAN2 or the indicated mutants probing for Hcp expression and secretion. RNAP is included as a lysis and loading control. The Western blot shown is representative of three independent experiments.

A previous report in Vibrio cholerae suggests that T6SS effectors may play a critical role in T6SS assembly (56). Thus, we tested whether AbCAN2 effectors Tse1 and Tse2 or VgrG2 were involved in T6SS inhibition. To this end, we generated mutant strains of AbCAN2 lacking *tse/tsi1*, *tse/tsi2*, *vgrG2*, or both *vgrGi* and *vgrG2* (*ΔvgrGi*,*2*) and determined their T6SS activity by Hcp secretion assay. We did not detect Hcp in the supernatant fraction of any of the mutant strains, indicating that VgrGi-mediated T6SS inhibition is independent of any proteins encoded within the two *vgrG* clusters of AbCAN2 (Fig. 3). Importantly, we also found that unlike *ΔvgrGi*, *ΔvgrG2*, and *ΔvgrGi*,*2* lack the ability to secrete Hcp and kill E. coli (Fig. 3 and Fig. S1b). Together, these results demonstrate that VgrG2 acts as a canonical VgrG; it is required for proper T6SS function and effector delivery into bacterial competitors.

**FIG 4:**
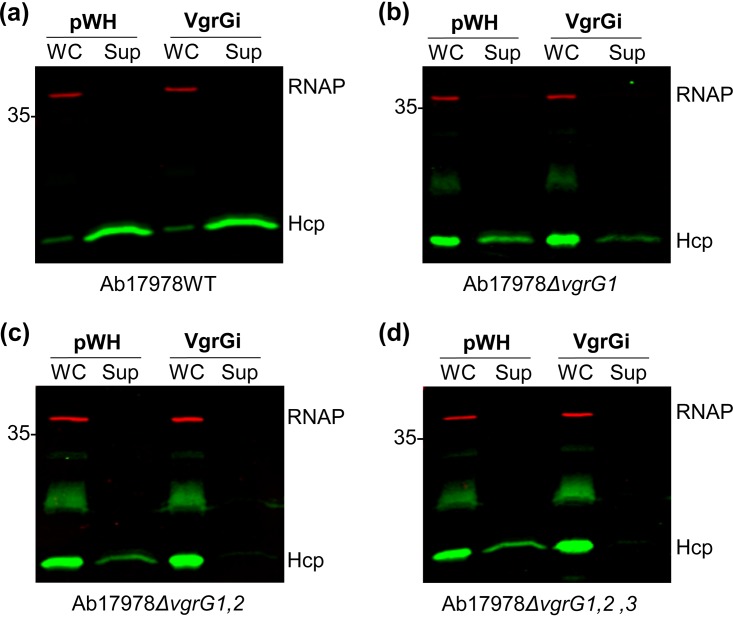
VgrGi inhibits the T6SS of VgrG mutant strains of Ab17978. Western blot analyses of OD-normalized whole-cell (WC) and supernatant (Sup) fractions of wild-type (WT) Ab17978 (a) or the mutant strains *ΔvgrG1* (b), *ΔvgrG1*,*2* (c) or *ΔvgrG1*,*2*,*3* (d) were performed, probing for Hcp expression and secretion. RNAP is included as a lysis and loading control. The Western blots shown are representative of three independent experiments.

Next, we tested whether VgrGi-mediated T6SS inhibition is due to an intrinsic property of AbCAN2 or whether VgrGi can also inhibit the T6SS of a heterologous host. To this end, we expressed plasmid-borne 6×His-tagged VgrGi in Ab17978 lacking LCP pAB3 (hereafter referred to as WT Ab17978), which possesses constitutive T6SS activity under standard laboratory conditions (40), and probed for Hcp secretion. We found that WT Ab17978 expressing VgrGi secreted similar levels of Hcp compared to the vector control (Fig. 4a and Fig. S2). This result suggested that VgrGi is unable to inhibit the T6SS of a noncognate host. Nonetheless, it is noteworthy that unlike AbCAN2, which encodes one canonical VgrG, VgrG2, Ab17978 encodes four VgrG homologs, each sufficient for assembling a functional T6SS (46). We reasoned that it was possible that VgrGi was unable to inhibit the T6SS of Ab17978 due to functional redundancy between the four VgrGs. Therefore, we then expressed VgrGi in mutant strains of Ab17978 lacking one to three VgrGs. We found that VgrGi expression in Ab17978*ΔvgrG1* resulted in a considerable reduction of Hcp detected in culture supernatants (Fig. 4b). Remarkably, VgrGi completely abolished Hcp secretion in Ab17978 *ΔvgrG1*,*2* and *ΔvgrG1*,*2*,*3* (Fig. 4c and d), indicating that the inhibitory capability of VgrGi extends beyond AbCAN2.

10.1128/mBio.02761-19.4 FIG S2 VgrGi expression in the indicated Ab17978 strains. Western blots of OD-normalized whole cell fractions of the indicated Ab17978 strains probing for 6×His-tagged VgrGi and RNAP (loading control). The Western blots shown are representative of three independent experiments. Download FIG S2, TIF file, 1.0 MB. Copyright © 2020 Lopez et al. 2020 Lopez et al. This content is distributed under the terms of the Creative Commons Attribution 4.0 International license.

Taken together, our results show that VgrGi can inhibit the T6SS of its native strain, as well as that of Ab17978 mutant strains with limited VgrGs. Given that AbCAN2 and Ab17978 possess a different arsenal of effectors, these findings provide further evidence that VgrGi-mediated T6SS inhibition is independent of the effectors expressed.

### VgrGi-mediated T6SS inhibition is due to a leucine-to-arginine mutation.

**FIG 5:**
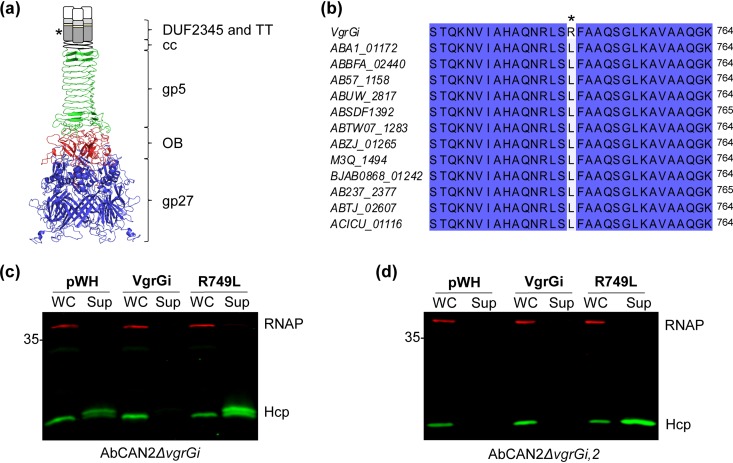
Arginine residue in DUF2345 domain of VgrGi is necessary for T6SS inhibition. (a) Schematic of the predicted structure of VgrGi based on the crystal structure of the P. aeruginosa VgrG1 (PDB 4MTK). Different domains are represented in different colors: gp27 (blue), OB-fold (red), and gp5 (green). The coiled-coil (cc), DUF2345 (dark gray rectangle), and transthyretin (TT; light gray rectangle) domains could not be modeled. Yellow and white rectangles represent areas for which no domain is predicted. The conceptualization of this figure borrowed heavily from Renault et al. (52). (b) Amino acid alignment of VgrGi with other class 1 VgrGs, according to the nomenclature of Fitzsimons et al. (58). Identical residues are highlighted in blue. In panels a and b, the asterisk highlights the L749R mutation present in VgrGi relative to other VgrGs of the same phylogenetic group. (c and d) Western blots probing for Hcp expression and secretion. OD-normalized whole-cell (WC) and supernatant (Sup) fractions of AbCAN2*ΔvgrGi* (c) or *ΔvgrGi*,*2* (d) expressing VgrGi, VgrGi R749L, or a vector control (pWH) are shown. RNAP was included as a lysis and loading control. The Western blots shown are representative of three independent experiments.

VgrGi is, to the best of our knowledge, the first VgrG homolog with an ability to inhibit the T6SS. Thus, we sought to identify unique characteristics that differentiate VgrGi from other VgrGs. However, protein sequence and structural prediction analyses revealed that VgrGi contains domains similar to those found in canonical VgrGs of diverse bacteria, such as VgrG1 of P. aeruginosa PAO1 (VgrG1^Pa^) and VgrG1 of EAEC 17-2 (VgrG1^Ec^) (52, 54, 57). (Despite their similar names, VgrG1^Pa^ and VgrG1^Ec^ are two distinct proteins.) VgrGi contains two domains at its N terminus that closely resemble bacteriophage T4 tail spike proteins gp27 (residues 1 to 426) and gp5 (residues 537 to 666) (Fig. 5a). These domains are linked by an oligosaccharide-binding (OB)-fold domain (residues 427 to 536). The C terminus of VgrGi is composed of a DUF2345 domain (residues 688 to 834), followed by a TT-like domain (residues 860 to 907) and a region of ∼130 residues with no predicted functional domains. Finally, the N- and C-terminal segments of VgrGi are linked by a predicted coiled-coil region (residues 667 to 687) (Fig. 5a).

Given that we identified no differences in the predicted functional domains of canonical VgrGs and those of VgrGi, we reasoned that VgrGi may possess critical differences in its primary sequence that are responsible for its inhibitory ability. VgrGs encoded by A. baumannii strains were recently grouped into six distinct phylogenetic groups (58). Based on the amino acid identity (>95%), we determined that VgrGi belongs to class 1 VgrGs. Interestingly, despite such remarkable levels of sequence conservation, we found that VgrGi contains a leucine-to-arginine substitution in position 749 compared to all other class 1 VgrGs (Fig. 5b). We hypothesized that the leucine-to-arginine mutation was responsible for the unprecedented ability of VgrGi to inhibit the T6SS of A. baumannii. To test our hypothesis, we expressed plasmid-borne 6×His-tagged VgrGi or a VgrGi revertant mutant containing an arginine-to-leucine mutation (hereafter referred to simply as R749L) in AbCAN2*ΔvgrGi* and probed for Hcp secretion. As expected, we detected Hcp in the supernatant of the vector control but not in the *vgrGi^+^* strain (Fig. 5c). Remarkably, *ΔvgrGi* expressing R749L (*vgrGi*_R749L_*^+^*) retained T6SS activity (Fig. 5c and Fig. S3a), indicating that the mutation R749L abrogated the inhibitory capability of VgrGi. In fact, *vgrGi*_R749L_*^+^* showed higher levels of Hcp secretion compared to the vector control (Fig. 5c), suggesting that R749L likely participates in T6SS assembly. To test this, we expressed plasmid-borne 6×His-tagged VgrGi or R749L in AbCAN2*ΔvgrGi*,*2*. This strain lacks T6SS activity (Fig. 3) but encodes all other genes necessary to assemble a functional T6SS. Thus, we hypothesized that if R749L participates in T6SS assembly, heterologous expression of this protein in *ΔvgrGi*,*2* would result in T6SS activity. Indeed, *ΔvgrGi*,*2* expressing R749L but not VgrGi or the empty vector secretes Hcp (Fig. 5d and Fig. S3b), indicating that the mutation R749L converts VgrGi to a canonical VgrG essential to T6SS assembly.

10.1128/mBio.02761-19.5 FIG S3 VgrGi/R749L expression in AbCAN2*ΔvgrGi* (a) or *ΔvgrGi*,*2* (b). Western blots of OD-normalized whole cell fractions of the indicated AbCAN2 strains probing for 6×His-tagged VgrGi and RNAP (loading control). The Western blots shown are representative of three independent experiments. Download FIG S3, TIF file, 0.6 MB. Copyright © 2020 Lopez et al. 2020 Lopez et al. This content is distributed under the terms of the Creative Commons Attribution 4.0 International license.

Altogether, our results indicate that VgrGi lacks any functional domains that differentiate it from canonical VgrGs. Instead, its inhibitory capability is due to a single amino acid mutation.

### Polar or charged residues within the SLFAAQ motif disrupt VgrG function.

**FIG 6:**
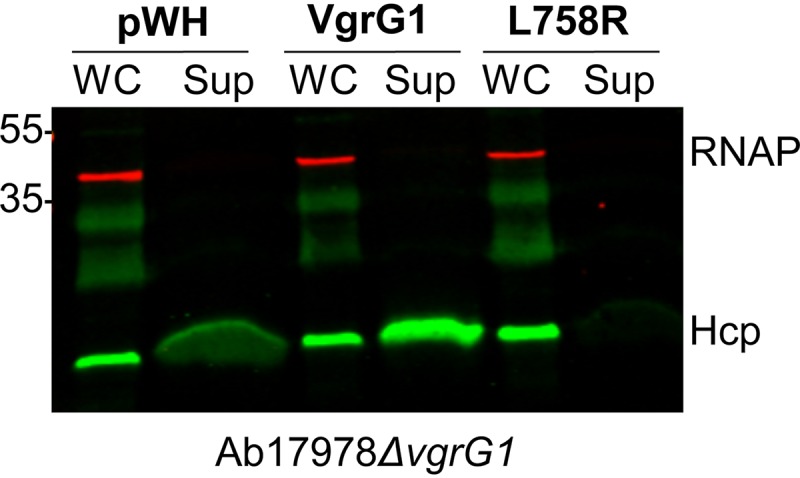
Leucine-to-arginine substitution converts canonical VgrG1 to an inhibitory VgrG. Western blot analysis of Ab17978*ΔvgrG1* expressing pWH (empty vector), VgrG1, or VgrG1 L758R probing for Hcp expression and secretion was performed. To facilitate the visualization of Hcp in the supernatant fractions of strains expressing pWH and L758R, twice the amount of supernatant from these strains (as determined by the OD) was loaded relative to Ab17978*ΔvgrG1* expressing VgrG1. RNAP was included as a lysis and loading control. The Western blot shown is representative of three independent experiments.

Our previous result suggests that a leucine residue in position 749 plays an important role in canonical VgrG function. A protein alignment analysis revealed that despite considerable levels of divergence among VgrGs of diverse A. baumannii strains, most VgrGs possess a conserved SLFAAQ motif (Fig. S4). The conservation of this motif further points to the leucine residue as important for VgrG function. Thus, we hypothesized that substitution of the conserved leucine for arginine would convert a canonical VgrG to an inhibitory VgrG. VgrG1 of Ab17978 (ACX60_17665) possesses a leucine residue within its SLFAAQ motif, L758 (Fig. S4), and is important for T6SS function (46). To test our hypothesis, we generated plasmid-borne 6×His-tagged VgrG1 or VgrG1 bearing a L758R mutation, expressed them in Ab17978*ΔvgrG1* (*vgrG1*^+^ and *vgrG1*_L758R_^+^, respectively) (Fig. S5), and determined the resulting levels of Hcp secretion. In contrast with the *vgrG1*^+^ strain, which had increased levels of Hcp secretion compared to the vector control, *vgrG1*_L758R_^+^, demonstrated a marginal level of Hcp secretion (Fig. 6). This result indicates that substitution of a conserved leucine residue for arginine confers on VgrG1 the ability to repress the T6SS of Ab17978.

10.1128/mBio.02761-19.6 FIG S4 Amino acid alignment of VgrGi with VgrGs representing diverse phylogenetic groups. Residues are highlighted based on identity, with dark blue indicating residues that are highly conserved. The line indicates the SLFAAQ motif, while the asterisk indicates the position of R749 in VgrGi. VgrG locus tags are colored according to their phylogenic group (Fitzsimons et al. [58]): class 1 (green), class 2 (blue), class 3 (yellow), class 4 (light orange), class 5 (pink), and class 6 (dark orange). ACX60_17665 did not group with any of the other homologs. Download FIG S4, TIF file, 1.8 MB. Copyright © 2020 Lopez et al. 2020 Lopez et al. This content is distributed under the terms of the Creative Commons Attribution 4.0 International license.

10.1128/mBio.02761-19.7 FIG S5 VgrG1/L758R expression by Ab17978*ΔvgrG1.* Western blot of OD-normalized whole cell fractions of Ab17978*ΔvgrG1* probing for 6xHis-tagged VgrG1 or VgrG1 L758R and RNAP (loading control). The Western blot shown is representative of three independent experiments. Download FIG S5, TIF file, 0.4 MB. Copyright © 2020 Lopez et al. 2020 Lopez et al. This content is distributed under the terms of the Creative Commons Attribution 4.0 International license.

**FIG 7:**
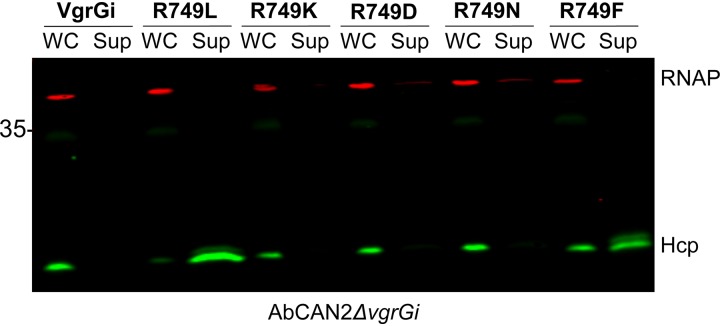
Polar and charged residues in position 749 result in T6SS inhibition by VgrGi. Western blot probing for Hcp expression and secretion was performed. OD-normalized whole-cell (WC) and supernatant (Sup) fractions of AbCAN2*ΔvgrGi* expressing the indicated VgrGi point mutants were assessed. RNAP was included as a lysis and loading control. The Western blot shown is representative of three independent experiments.

Next, we tested whether VgrGi-mediated T6SS inhibition depends on a specific property of arginine or whether other amino acids yield similar phenotypes. To this end, we generated pWH1266 vector constructs containing the following point mutants of VgrGi: R749K, R749D, R749N, and R749F. Then, we expressed these VgrGi variants in AbCAN2*ΔvgrGi* (Fig. S6) and determined whether expression of these constructs results in T6SS inhibition. We found that VgrGi variants containing polar or charged residues (i.e., R749K, R749D, and R749N) inhibit Hcp secretion (Fig. 7). In contrast, VgrGi variants containing nonpolar residues (i.e., R749L and R749F) do not inhibit Hcp secretion (Fig. 7). Similar results were obtained when these constructs were expressed in Ab17978*ΔvgrG1*,*2* (Fig. S7). Collectively, our results indicate that polar or charged residues in the second position of the SLFAAQ motif disrupt the canonical function of VgrGs in T6SS assembly.

10.1128/mBio.02761-19.8 FIG S6 Expression of the indicated VgrGi point mutants by AbCAN2*ΔvgrGi*. Western blot of OD-normalized whole cell fractions of AbCAN2*ΔvgrGi* probing for the indicated 6×His-tagged VgrGi variants and RNAP (loading control). The Western blot shown is representative of three independent experiments. Download FIG S6, TIF file, 0.8 MB. Copyright © 2020 Lopez et al. 2020 Lopez et al. This content is distributed under the terms of the Creative Commons Attribution 4.0 International license.

10.1128/mBio.02761-19.9 FIG S7 Polar and charged residues in position 749 result in T6SS inhibition by VgrGi. (a) Western blot probing for Hcp expression and secretion. Shown are OD-normalized whole cell (WC) and supernatant (Sup) fractions of Ab17978*ΔvgrG1*,*2* expressing the indicated VgrGi point mutants. (b) Western blot of OD-normalized whole cell (WC) fractions of Ab17978*ΔvgrG1*,*2* probing for the indicated 6×His-tagged VgrGi variants. RNAP is included as a lysis and/or loading control. The Western blots shown are representative of three independent experiments. Download FIG S7, TIF file, 1.6 MB. Copyright © 2020 Lopez et al. 2020 Lopez et al. This content is distributed under the terms of the Creative Commons Attribution 4.0 International license.

### The C terminus of VgrG is essential to T6SS function in *A. baumannii*.

**FIG 8:**
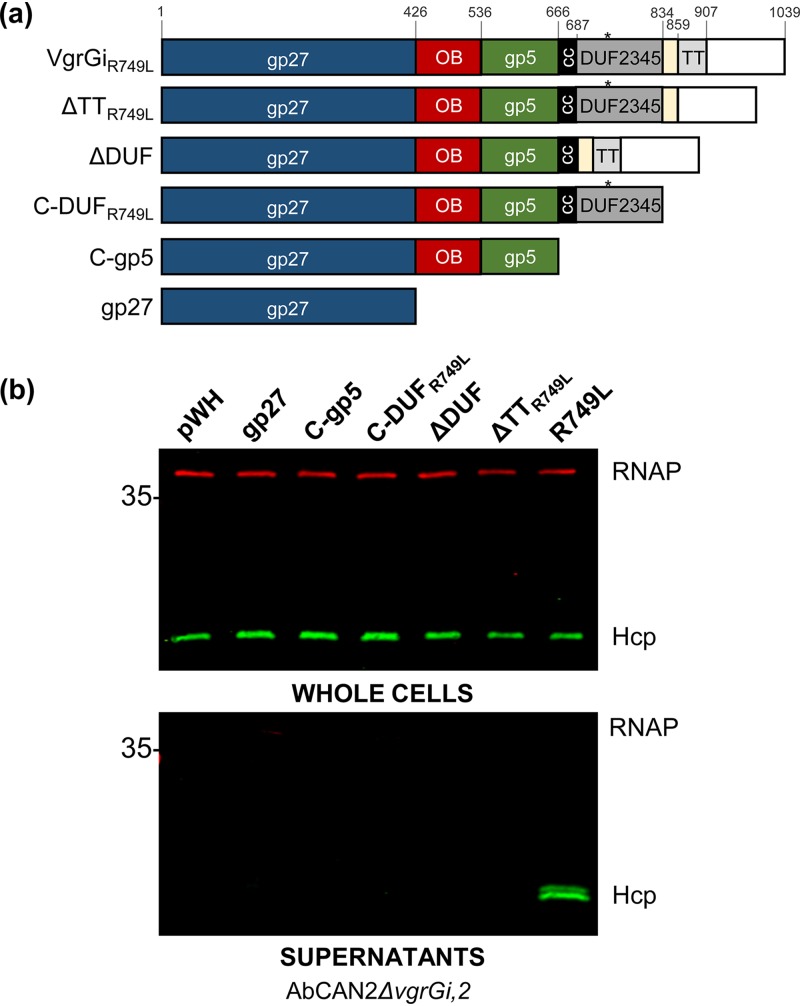
The C terminus of VgrGi_R749L_ is required for T6SS activity. (a) Schematic of the domains present in VgrGi_R749L_, as well as the truncations made for the experiment in panel b. The amino acid boundaries are indicated, and the color scheme is consistent with that of Fig. 5a. The asterisk indicates the position of the R749L mutation. (b) Western blot analyses probing for Hcp expression (top) and secretion (bottom) in AbCAN2*ΔvgrGi*,*2* expressing the indicated VgrGi_R749L_ truncations were carried out. RNAP was included as a lysis and loading control. The Western blots shown are representative of three independent experiments.

The SLFAAQ motif lies within the C-terminal DUF2345 domain of VgrGi. Although present in VgrGs of diverse Gram-negative bacteria, the role of the DUF2345 domain in T6SS assembly remains poorly understood. Recent work in E. coli showed that the gp27 domain of VgrG1^Ec^ is sufficient for T6SS activity, indicating that the DUF2345 and TT domains are dispensable for T6SS assembly (52). Similarly, the C terminus of P. aeruginosa VgrG5 is not required to support VgrG4b secretion (53). Nonetheless, our results indicate that a point mutation within the C-terminal DUF2345 domain of A. baumannii VgrGs is sufficient to disrupt T6SS activity. Thus, we hypothesized that in A. baumannii, the DUF2345 domain plays an essential role in the assembly of a functional T6SS. To test our hypothesis, we expressed different truncations of R749L in AbCAN2*ΔvgrGi*,*2* and determined the resulting levels of Hcp secretion. As shown in Figure 5d, *ΔvgrGi*,*2* lacks T6SS activity unless it expresses a functional VgrG (e.g., full-length R749L). The R749L constructs tested include the gp27 domain (gp27), a C-terminal truncation ending with the gp5 domain (C-gp5), a C-terminal truncation ending with the DUF2345 domain (C-DUF_R749L_), and mutants lacking either the DUF2345 domain (ΔDUF) or the TT domain (ΔTT_R749L_) but possessing all other domains (Fig. 8a and Fig. S8). We found that in contrast to full-length R749L, the expression of none of the R749L truncations tested led to Hcp secretion (Fig. 8b). This result indicates that the C terminus of R749L is essential to the role of VgrG in assembling a functional T6SS.

10.1128/mBio.02761-19.10 FIG S8 Expression of the indicated VgrGi_R749L_ truncations in AbCAN2*ΔvgrGi*,*2.* Western blots of OD-normalized whole cell fractions of AbCAN2*ΔvgrGi*,*2* probing for the indicated 6×His-tagged VgrGi_R749L_ truncations and RNAP (loading control). The Western blot shown is representative of three independent experiments. Download FIG S8, TIF file, 0.7 MB. Copyright © 2020 Lopez et al. 2020 Lopez et al. This content is distributed under the terms of the Creative Commons Attribution 4.0 International license.

## DISCUSSION

The T6SS is a critical weapon for interbacterial warfare (12). Seminal studies have provided general principles that are largely applicable to the T6SS of various bacterial species. However, it is becoming increasingly clear that the T6SS is a diversified machine, with important structural and functional differences depending on the bacterial host (31, 59, 60). Here, we identified a VgrG homolog with an unprecedented ability to inhibit the T6SS of the clinical isolate AbCAN2. Our efforts to characterize the mechanism underlying VgrGi-mediated T6SS inhibition led us to determine that the C terminus of VgrG is essential for functional T6SS assembly in A. baumannii.

VgrG is one of the most versatile proteins of the T6SS. It is an essential structural component of the baseplate and sharpens the Hcp tube to facilitate penetration into adjacent cells and delivery of effectors (21, 22, 54). Despite its relevance, VgrG remains largely understudied compared to other T6SS proteins, likely due to its inherent insolubility (61). Only one full-length VgrG protein, VgrG1^Pa^, has been structurally characterized (57). Notably, VgrG1^Pa^ is much shorter compared to other VgrGs (643 amino acids [aa] versus 841 aa [VgrG1^Ec^] and 1,039 aa [VgrGi]). Thus, the functional domains present in longer VgrGs remain largely uncharacterized.

One of the functional domains present in longer VgrGs is the DUF2345 domain. In Escherichia coli, this domain has been shown to stabilize the interaction between the TT domain of VgrG1 and effector Tle1, but it is dispensable for T6SS assembly and dynamics (52, 54). Despite its seeming dispensability for T6SS assembly, this domain is present in the VgrGs of a wide range of Gram-negative bacteria, including V. cholerae (VCV52_2925), Klebsiella pneumoniae (BN49_3373), P. aeruginosa (PA0262), and Salmonella enterica (FJR63_23580). In fact, VgrGs were originally described as possessing a COG4253 functional domain, which belongs to the DUF2345 superfamily (60). Our results suggest that the DUF2345 domain may play a role in T6SS assembly in A. baumannii. However, since C-DUF_R749L_ is not sufficient for T6SS assembly (Fig. 8), the DUF2345 domain may require additional elements present at the C terminus of VgrG for proper function. Moreover, a recent bioinformatic analysis of 73 VgrGs across 22 A. baumannii strains, as well as the soil bacterium A. baylyi ADP1, showed that all VgrGs contain a DUF2345 domain (58). The high levels of conservation suggest that the DUF2345 domain may play a particularly important role in the T6SS of Acinetobacter species.

It is noteworthy that our results are not in conflict with previous results indicating that the C terminus of VgrG is dispensable for T6SS function (52, 53). Instead, we propose that over evolutionary time, bacteria developed changes to their T6SS architecture, leading to specialized systems. Indeed, it was recently reported that TssA homologs carry out three diverse functions depending on their C-terminal domain (62). Moreover, homologs of membrane complex protein TssM possess a Walker A motif; however, no general role for this motif has been described. In Agrobacterium tumefaciens, for instance, TssM exhibits ATP binding and hydrolysis, as well as ATP-binding-dependent conformational changes, all of which are implicated in T6SS function (63, 64). In contrast, the Walker A motif of the Edwardsiella tarda TssM, is dispensable for T6SS activity (65). Thus, it seems likely that bacteria have co-opted conserved structural proteins to better suit their needs.

Future work will characterize the mechanism by which VgrGi inhibits the T6SS, since it may provide insight into the role of the C terminus of A. baumannii VgrGs. Our finding that VgrGi inhibits the T6SS of Ab17978 only when endogenous VgrGs are limited suggests that VgrGi acts at the posttranslational level. We propose that VgrGi likely inhibits the T6SS by preventing its assembly. VgrGs can form heterotrimeric assemblies (21, 53, 66, 67). Thus, it is possible that VgrGi binds canonical VgrGs and prevents essential protein-protein interactions with other structural components of the T6SS. PAAR proteins, for instance, bind VgrG proteins at their C termini (68). In A. baylyi, PAAR proteins are essential for effective T6SS firing (68). Thus, VgrG trimers containing VgrGi may be unable to bind PAAR proteins, which would be expected to disrupt T6SS assembly. Moreover, it is possible that VgrGi possesses conformational changes that abrogate critical baseplate-membrane complex interactions. Alternatively, it is possible that VgrGi acts post-T6SS assembly. The T6SS is expected to undergo two large conformational changes to enable deployment of the spiked tube. First, conformational changes in the baseplate are proposed to trigger sheath contraction (22, 69, 70). Second, the membrane complex is expected to undergo large conformational changes to enable the passage of the spiked tube (19, 71). It is possible that a leucine-to-arginine mutation causes aberrant interactions between VgrG and components of the membrane complex or baseplate, which prevent the necessary conformational changes for T6SS firing. In this instance, VgrGi may prove to be an invaluable tool to lock the dynamic T6SS in a static conformation and facilitate the structural characterization of the Acinetobacter T6SS.

Finally, it is noteworthy that we found an inactivating mutation of the T6SS in a clinical isolate. Although we cannot say with certainty whether this mutation was acquired during culturing or in the human host, it is tempting to speculate that there may be a selective pressure against the T6SS of medically relevant strains. As previously mentioned, the T6SS is an energetically expensive machine. Thus, there may be benefits to inactivating this machine, for example, to conserve energy. Moreover, T6SS structural components have been shown to be immunogenic (13, 72). It is possible that T6SS-inactive strains are selected for in the human host, since they are more likely to bypass detection by the immune system. In fact, most of the characterized virulence factors of A. baumannii involve evading immune detection or overcoming nutritional immunity (38). Thus, eliminating the T6SS may represent yet another strategy to bypass immune detection. Nonetheless, the T6SS confers a competitive advantage to nonpathogenic bacteria living in polymicrobial communities. Thus, it is likely that the environment exerts selective pressure in favor of a silenced T6SS in clinical A. baumannii strains and a constitutively active T6SS in nonpathogenic strains, as was previously proposed for V. cholerae (29). Indeed, clinical isolates of A. baumannii often have a nonfunctional T6SS due to genetic disruptions or absence of T6SS genes (44, 73–75). Our work indicates that point mutations at critical residues of T6SS structural proteins could constitute an underappreciated mechanism by which the T6SS is genetically disrupted.

Collectively, we have demonstrated that unlike in other bacteria, the C terminus of VgrG is essential to functional T6SS assembly in A. baumannii. The specific roles of the DUF2345 and other C-terminal domains of VgrGs warrant further investigation.

## MATERIALS AND METHODS

### Bacterial strains and growth conditions.

All strains and plasmids used in this study are listed in Table S2. Strains were grown in Luria-Bertani (LB) broth at 37°C with shaking. Antibiotics were added to the media when appropriate (see below).

10.1128/mBio.02761-19.2 TABLE S2 Strains, plasmids. and primers used in this study. Download Table S2, DOCX file, 0.02 MB. Copyright © 2020 Lopez et al. 2020 Lopez et al. This content is distributed under the terms of the Creative Commons Attribution 4.0 International license.

### Transposon mutagenesis and screen T6SS-active mutants.

Our method for transposon mutagenesis has been described previously (41). Plasmid pSAM::OmpAp+Tn903 was introduced to AbCAN2 from E. coli BW19851 by biparental mating. Briefly, overnight cultures were pelleted, washed three times with fresh LB medium, and resuspended at an optical density at 600 nm (OD_600_) of 1.0. The cultures were then mixed at a 1:1 ratio and spotted onto a dry LB agar plate. After a 5-h incubation at 37°C, the spot was resuspended in 1 ml of LB broth, and 10-fold dilutions were plated on LB agar containing kanamycin (20 μg/ml) plus chloramphenicol (12.5 μg/ml), followed by incubation overnight at 37°C. The transposon mutants obtained were subsequently subjected to a colony blot (51) to identify mutants with an active T6SS. Briefly, colonies were transferred to a nitrocellulose membrane and allowed to dry at room temperature for 30 min. The dried membrane is washed twice with Tris-buffered saline–Tween and treated as a normal Western blot, probing for Hcp and RNAP (see below). Candidates were then isolated and verified using a standard Hcp secretion assay (see below).

Identification of transposon insertion sites was carried out using a method adapted from Goodman et al. (76). Transposon mutant gDNA was digested with MmeI and separated on a 0.7% agarose gel by electrophoresis. Fragments ∼500 and ∼1,600 bp in length were extracted and appended with double-stranded oligonucleotide sequencing adapters by ligation. Finally, the transposon plus two 16-bp genomic sequences flanking the transposon was amplified by PCR and sequenced. The insertion site was verified by amplifying a genomic fragment of the putative insertion site by PCR and sequencing that fragment as well.

### Hcp secretion assay and Western blot analysis.

Overnight cultures were back-diluted in fresh LB medium to an OD_600_ of 0.025 and grown at 37°C with shaking until they reached an OD_600_ of 0.35 to 0.5 (AbCAN2) or 0.4 to 0.7 (Ab17978). The cells were then pelleted by centrifugation. The cells were resuspended in Laemmli buffer to a final OD_600_ of 10, while the supernatant fraction was centrifuged once again (as above) to pellet residual cells. Supernatant proteins were subsequently precipitated with trichloroacetic acid, as previously described (39). Optical desity-normalized volumes of whole cells or supernatants were loaded onto 15% (for Hcp) or 8% (for VgrGs) SDS-PAGE gels for separation, transferred to a nitrocellulose membrane, and probed with polyclonal rabbit anti-Hcp (1:1,000) (39), polyclonal rabbit anti-6×His (1:2,000; Invitrogen, Waltham, MA) or monoclonal mouse anti-RNA polymerase (1:2,600; BioLegend, San Diego, CA). Western blots were then probed with IRDye-conjugated anti-mouse and anti-rabbit secondary antibodies (both at 1:15,000; LI-COR Biosciences, Lincoln, NE) and visualized with an Odyssey CLx imaging system (LI-COR Biosciences).

### Generation of mutants and pWH constructs.

The primers used in this study are listed in Table S2. Marked mutant strains were generated by substitution of the gene of interest by a kanamycin resistance marker, as described previously (77). Selection was carried out using kanamycin (30 μg/ml). To generate clean mutants, electrocompetent marked mutants were transformed with pAT03 to remove the FRT-flanked kanamycin resistance cassette. Transformants were plated on LB agar containing 2 mM IPTG (isopropyl-β-d-thiogalactopyranoside) plus hygromycin (600 μg/ml). Mutant strains were verified by PCR and sequencing.

Constructs pWH-*vgrGi*-6×His and pWH-*vgrG1*-6×His were generated by restriction cloning using the EcoRI and PstI sites. These plasmids were then used as templates to make point mutants of VgrGi and VgrG1, respectively. Specifically, point mutants were generated using the QuikChange II site-directed mutagenesis kit (Agilent Technologies, Santa Clara, CA) according to the manufacturer’s instructions. Constructs of VgrGi truncations ΔDUF and ΔTT_R749L_ were generated by inverse PCR of pWH-*vgrGi*-6×His, followed by blunt ligation. All other VgrGi truncation constructs were generated by restriction cloning, as described above. Selection was carried out using tetracycline (15 μg/ml for AbCAN2 and 10 μg/ml for Ab17978). All constructs were verified by PCR and sequencing.

### Bacterial killing assay.

Overnight cultures were washed three times with fresh LB and normalized to an OD_600_ of 1. Predator and prey strains were mixed at a 10:1 ratio, respectively, and spotted on dry LB agar. After a 3.5-h incubation at 37°C, spots were resuspended in LB medium, and serial dilutions were spotted on LB agar with the appropriate antibiotic (kanamycin 50 μg/ml for E. coli MG1655R/pBAV-*gfp* and tetracycline 15 μg/ml for E. coli HB101/pWH1266). The CFU of surviving prey cells were enumerated after overnight incubation at 37°C.

### VgrGi structural model and alignments.

VgrGi C-gp5 (residues 1 to 666) was submitted to the iterative threading assembly refinement (I-TASSER) server (78), using the crystal structure of P. aeruginosa PAO1 VgrG1 (PDB 4MTK) as a template without alignment. The trimer assembly was constructed by aligning the VgrGi model to each VgrG1^Pa^ monomer in Biological Assembly 1 (PDB 4MTK), using the PyMOL Molecular Graphics System (v1.2r3pre; Schrödinger, LLC).

Domain designations were made according to Interpro (79) and HHPred (80) servers. The predictions for the coiled-coil domain and the DUF2345 domain overlap in residues 668 to 697; however, for the construction of the VgrGi truncations, preference was given to the DUF2345 domain. Sequence alignments were done using Clustal Omega (81).

### Data availability.

The genome of AbCAN2 (formerly named Ab1225) was deposited to GenBank under accession number CP045428.
