# Supplementary material for: The Tip of the VgrG Spike Is Essential to Functional Type VI Secretion System Assembly in Acinetobacter baumannii
Source: mBio. 2020 Jan 14;11(1):e02761-19. doi: 10.1128/mBio.02761-19 (PMC6960284; doi:10.1128/mBio.02761-19)
Supplement: TABLE S1 [file mBio.02761-19-st001.docx]

**Table S1.** Comparison between T6SS-related genes of AbCAN2 and Ab17978

| **Gene name^#^** | **Length (bp) Ab17978/AbCAN2** | **Cover** | **Identity** |
| --- | --- | --- | --- |
| ACX60_11685 | 693 | 100 | 99 |
| TssB | 504 | 100 | 99 |
| TssC | 1482 | 100 | 99 |
| Hcp | 504 | 100 | 99 |
| TssE | 477 | 100 | 100 |
| TssF | 1812 | 100 | 99 |
| TssG | 999 | 100 | 99 |
| ACX60_11650 | 1413 | 100 | 98 |
| TssM | 3824 | 100 | 99 |
| TagF | 960 | 100 | 99 |
| TagN | 768 | 100 | 99 |
| PAAR | 264 | 100 | 99 |
| ClpV | 2682 | 100 | 98 |
| TssA | 1098/1095 | 99 | 99 |
| TssK | 1365 | 100 | 99 |
| TssL | 807 | 100 | 99 |
| ACX60_11605 | 603 | 100 | 99 |
| TagX | 954 | 100 | 99 |
| ^#^ hypothetical proteins are named based on the locus tag of Ab17978 | | | |
