# Supplementary material for: The Tip of the VgrG Spike Is Essential to Functional Type VI Secretion System Assembly in Acinetobacter baumannii
Source: mBio. 2020 Jan 14;11(1):e02761-19. doi: 10.1128/mBio.02761-19 (PMC6960284; doi:10.1128/mBio.02761-19)
Supplement: TABLE S2 [file mBio.02761-19-st002.docx]

**Table S2.** Strains, plasmids and primers used in this study

| **Plasmid or strain** | **Description** | **Reference** |
| --- | --- | --- |
| **Strains** |  |  |
| AbCAN2 | Coccygeal isolate | (40) |
| AbCAN2 F1A | Transposon insertion mutant of *vgrGi* | This study |
| AbCAN2 G9 | Transposon insertion mutant of *vgrGi* | This study |
| AbCAN2 G31 | Transposon insertion mutant of *vgrGi* | This study |
| AbCAN2 G32 | Transposon insertion mutant of *vgrGi* | This study |
| AbCAN2*ΔvgrGi* | Clean deletion mutant of *vgrGi* | This study |
| AbCAN2*vgrGi+* | AbCAN2*ΔvgrGi* expressing pWH-*vgrGi*-6xHis | This study |
| AbCAN2*vgrGi*_R749L_*+* | AbCAN2*ΔvgrGi* expressing pWH-*R749L*-6xHis | This study |
| AbCAN2*ΔvgrG2* | Kanamycin cassette-marked deletion mutant of *vgrG2* | This study |
| AbCAN2*ΔvgrGi,2* | Clean deletion mutant of *vgrGi* with a kanamycin cassette-marked deletion of *vgrG2* | This study |
| AbCAN2*Δtse/tsi1* | Kanamycin cassette-marked deletion mutant of *tse1* and *tsi1* | This study |
| AbCAN2*Δtse/tsi2* | Kanamycin cassette-marked deletion mutant of *tse2* and *tsi2* | This study |
| Ab17978*ΔvgrG1* | Clean deletion mutant of *vgrG1* | (47) |
| Ab17978*ΔvgrG1,2* | Clean deletion mutant of *vgrG1* and *vgrG2* | (47) |
| Ab17978*ΔvgrG1,2,3* | Clean deletion mutant of *vgrG1, vgrG2* and *vgrG3* | (47) |
| *E. coli* MG1655R pBAV-*gfp* | Used as prey for killing assay | (41) |
| *E. coli* HB101 pWH1266 | Used as prey for killing assay |  |
| **Plasmids** |  |  |
| pWH1266 (pWH) | *Acinetobacter-E. coli* shuttle vector | (84) |
| pWH-*vgrGi*-6xHis | pWH containing C-terminally 6xHis-tagged AbCAN2 *vgrGi* under the control of its predicted native promoter | This study |
| pWH-*R749L*-6xHis | pWH-*vgrGi*-6xHis with mutation R749L | This study |
| pWH-*R749K*-6xHis | pWH-*vgrGi*-6xHis with mutation R749K | This study |
| pWH-*R749D*-6xHis | pWH-*vgrGi*-6xHis with mutation R749D | This study |
| pWH-*R749N*-6xHis | pWH-*vgrGi*-6xHis with mutation R749N | This study |
| pWH-*R749F*-6xHis | pWH-*vgrGi*-6xHis with mutation R749F | This study |
| pWH-*vgrG1*-6xHis | pWH containing C-terminally 6xHis-tagged Ab17978 *vgrG1* under the control of its predicted native promoter | This study |
| pWH-*L758R*-6xHis | pWH-*vgrG1*-6xHis with mutation L758R | This study |
| pWH-gp27-6xHis | gp27 domain of *vgrGi* cloned into pWH; truncation contains C-terminal 6xHis tag and its expression is driven by the predicted native promoter of *vgrGi* | This study |
| pWH-C-gp5-6xHis | C-terminal truncation of *vgrGi* ending in gp5 cloned into pWH; truncation contains C-terminal 6xHis tag and its expression is driven by the predicted native promoter of *vgrGi* | This study |
| pWH-C-DUF2345-R749L-6xHis | C-terminal truncation of *vgrGi* ending in DUF2345 cloned into pWH; truncation contains C-terminal 6xHis tag and its expression is driven by the predicted native promoter of *vgrGi* | This study |
| pWH-delDUF-6xHis | pWH-*vgrGi*-6xHis with *vgrGi* lacking its DUF2345 domain | This study |
| pWH-delTT-6xHis | pWH-*R749L*-6xHis with *R749L* lacking its TT-like domain | This study |
| **Primer name** | **Sequence (5’-3’)** | **Description** |
| A31-MmeI_adap_T | TTCCTTACACGACGCTCTTCCGATCTNN (top strand)  AGATCGGAAGAGCGTCGTGTAAGGAA (bottom strand) | Double-stranded oligonucleotide sequencing adapter with an NN overhang at the 3’ end of the top strand. |
| A32–MmeI_adap_PCR | CGACCACCGAACACTCTTTCCTTACACGACGCTCTTCCGATCT | Extend adaptor sequence to promote higher-quality sequence reads at the 5’ end |
| A33–Mar_PCR_5 | GACTTGACGGGACGGCGGCTTTG | Extend adaptor sequence to promote higher-quality sequence reads at the 5’ end |
| A34–Mar_PCR_3 | CGAGAGTAGGGAACTGCCAGG | Extend adaptor sequence to promote higher-quality sequence reads at the 3’ end. Also used for sequencing. |
| A36–Adapt_B_Pcr | TTACTATGCCGCTGGTGGCTTGTGAGATTCCTTACACGACGCTCTTCCGATCT | Extend adaptor sequence to promote higher-quality sequence reads at the 3’ end |
| A38–Adapt_T_Seq2 | CGACCACCGAACACTCTTTC | Use with primer A33 to amplify sequence at the 5’ end of transposon. Also used for sequencing. |
| A37–Adapt_B_seq | TTACTATGCCGCTGGTGGCT | Use with primer A34 to amplify sequence at the 3’ end of transposon. Also used for sequencing. |
| A41-Mar_Pcr_5v2 | CTGCAGGTCTCGAGGGCG | Used for sequencing genomic region at 5’ end of transposon |
| A59-VgrGi-FRT-F | CTCACAGCATCGTTCGGTGTATCTCCAATTTTCAGATGCATCCCTGAATAGTCAGGTTTTTTTACAACGTATAGATGGGCAACATTATCTTAATCAGGGCGCGATTGTGTAGGCTGGAGCTGCTTC | Generate AbCAN2*ΔvgrGi* by recombineering |
| A60-VgrGi-FRT-R | TTCTTTCTGTTGTGTATTCTCTTTAGCCATCAATATAAAACCTTTAATTCATTCAATTGCAGCAATATTTTTCTAAGTTGAATCGCCCTATTCATCCAAAGACATATGAATATCCTCCTTAGTTCCTATTCCG | Generate AbCAN2*ΔvgrGi* by recombineering |
| A79-Tsei-FRT-F | TTAGATACTTTTGTACGGTCAAAAAAGTAGTTTGGATGAATAGGGCGATTCAACTTAGAAAAATATTGCTGCAATTGAATGAATTAAAGGTTTTATATTGGCGATTGTGTAGGCTGGAGCTGCTTC | Generate AbCAN2*Δtsei,tsii* by recombineering |
| A77-Tsii-FRT-R | TTATAGATCACAGATGTAAATAATCATAAAAAAATAATACCTCTACAAGCCTGTCAATAAAATTATTTCTCATTAAAAGCTAAAATTTTAATTCGGTTTATCATATGAATATCCTCCTTAGTTCCTATTCCG | Generate AbCAN2*Δtsei,tsii* by recombineering |
| A97-VgrG2-FRT-F | ATAAGAAGTGTCTCTTACACTTAAAAATCACTGCTAATGAAATAGACAGATTTGTATGTATTTTATAAAATACTGCAAAATATAATTTAGTCCTATAAAGGCGATTGTGTAGGCTGGAGCTGCTTC | Generate AbCAN2*ΔvgrG2* by recombineering |
| A98-VgrG2-FRT-R | TACTACCGCGACCTGTCCGGTATTTGTTCTCACACTTGGCTTTGCTTTCGCTTGAGAAATAGTTTTTGGTGCTGTTGGCATTTTTATAAATTCTCAATTCCATATGAATATCCTCCTTAGTTCCTATTCCG | Generate AbCAN2*ΔvgrG2* by recombineering |
| 69-Mutant tse2,tsi2 F | GTAAATCCATTAAGGGCTCAAGACTCGAAAAATACAGAGACTGCTCTTAATCCTATGAGTTTTAAGTTTAAATAAGAATTGAGAATTTATAAAAGCGATTGTGTAGGCTGGAGCTGCTTCG | Generate AbCAN2*Δtse2,tsi2* by recombineering |
| 70-Mutant tse2,tsi2 R | CTTACCTATTGATCAAAATATTACCCTTACAAAAAATATCGACCTGATAAGTTTAAGATTATCTAAATAGAGATGTTCTTAAAAACTCTAAAAACATATGAATATCCTCCTTAGTTCCTATTCCG | Generate AbCAN2*Δtse2,tsi2* by recombineering |
| A46-VGi-EcoRIF | ATATGAATTCATGGGCTATTAAATTATTGAC | Clone AbCAN2 *vgrGi*-6xHis into pWH |
| A64-VGi6his-PstIR | ACTTCTGCAGCTAGTGGTGGTGGTGGTGGTGCTTTTTTGACCGTACA | Clone AbCAN2 *vgrGi*-6xHis into pWH |
| 79-VgrGi R749L 1 | CACTTTGTGCTGCAAAGAGGCTGAGCCTATTTTGG | R749L substitution of pWH-*vgrGi*-6xHis |
| 80-VgrGi R749L 2 | CCAAAATAGGCTCAGCCTCTTTGCAGCACAAAGTG | R749L substitution of pWH-*vgrGi*-6xHis |
| 127-VgrGi R749K 1 | TAGTCCACTTTGTGCTGCAAACTTGCTGAGCCTATTTTGGGCATG | R749K substitution of pWH-*vgrGi*-6xHis |
| 128-VgrGi R749K 2 | CATGCCCAAAATAGGCTCAGCAAGTTTGCAGCACAAAGTGGACTA | R749K substitution of pWH-*vgrGi*-6xHis |
| 129-VgrGi R749D 1 | CCACTTTGTGCTGCAAAGTCGCTGAGCCTATTTTGGGC | R749D substitution of pWH-*vgrGi*-6xHis |
| 130-VgrGi R749D 2 | GCCCAAAATAGGCTCAGCGACTTTGCAGCACAAAGTGG | R749D substitution of pWH-*vgrGi*-6xHis |
| 135-VgrGi R749N 1 | CCACTTTGTGCTGCAAAGTTGCTGAGCCTATTTTGGGC | R749N substitution of pWH-*vgrGi*-6xHis |
| 136-VgrGi R749N 2 | GCCCAAAATAGGCTCAGCAACTTTGCAGCACAAAGTGG | R749N substitution of pWH-*vgrGi*-6xHis |
| 137-VgrGi R749F 1 | CCACTTTGTGCTGCAAAGAAGCTGAGCCTATTTTGGGC | R749F substitution of pWH-*vgrGi*-6xHis |
| 138-VgrGi R749F 2 | GCCCAAAATAGGCTCAGCTTCTTTGCAGCACAAAGTGG | R749F substitution of pWH-*vgrGi*-6xHis |
| 235-PromVgrG1 FW EcoRI Ab17978 | ATATGAATTCGATGCCAAGATGATATTGCGC | Clone Ab17978 *vgrG1*-6xHis into pWH |
| 236-VgrG1 His RV PstI Ab17978 | ATCTGCAGTCAGTGGTGATGGTGATGATGATTTACCACATGTATTTTATATTGCTTATTC | Clone Ab17978 *vgrG1*-6xHis into pWH |
| 81-VrgG1 17978 L758R 1 | CCTTGTTGGGCTGCAAAACGGCTAATCTTATTTTGGG | L758R substitution of pWH-*vgrG1*-6xHis |
| 82-VrgG1 17978 L758R 2 | CCCAAAATAAGATTAGCCGTTTTGCAGCCCAACAAGG | L758R substitution of pWH-*vgrG1*-6xHis |
| 276-VgrGipromFW EcoRI | ATATGAATTCATGGGCTATTAAATTATTGACGATC | Use with primers 277-279 to generate the specified truncations of *vgrGi_R749L_* and insert them into pWH1266 by restriction cloning |
| 277-gp27hisRV PstI | ATATCTGCAGCTAGTGGTGGTGGTGGTGGTGTGGTGTTGTTGGAATATAACG | Generate pWH-gp27-6xHis |
| 278-gp5HisRV PstI | ATATCTGCAGCTAGTGGTGGTGGTGGTGGTGAGCAATGTCACTCAGTGC | Generate pWH-C-gp5-6xHis |
| 279-DUFHisRV PstI | ATATCTGCAGCTAGTGGTGGTGGTGGTGGTGTGCCATAAACAAATGCTGTC | Generate pWH-C-DUF2345-R749L-6xHis |
| 270-deleteDUFRV | AATTTGTGAGGCAAAATCTTTTAACTGTTC | Generate pWH-delDUF-6xHis by inverse PCR |
| 271-deleteDUFFW | GGAGCAAGTGCAAACGCC | Generate pWH-delDUF-6xHis by inverse PCR |
| 272-deleteTTRV | CCGAAGTAGCTCCAATGCTCC | Generate pWH-delTT-6xHis by inverse PCR |
| 273-deleteTTFW | AAGGATAATACAAGTGCATGGCTACAAAG | Generate pWH-delTT-6xHis by inverse PCR |
